# The value of artificial intelligence in ultrasound imaging for predicting molecular subtypes of breast cancer: a meta-analysis

**DOI:** 10.3389/fonc.2026.1748473

**Published:** 2026-03-12

**Authors:** Yu-Hang Cheng, Jian Dong, Zhen Wang, Huan Zhao, Ming Chen, Ting Ma

**Affiliations:** 1 Department of Ultrasound Medicine, the First Affiliated Hospital of Shihezi University, Shihezi, Xinjiang, China; 2 Department of Medical Ultrasound, Tongji Hospital, Tongji Medical College, Huazhong University of Science and Technology, Wuhan, China

**Keywords:** artificial intelligence, breast cancer, convolutional neural network, deep learning, molecular subtypes

## Abstract

**Background:**

The study aims to integrate an evaluation of the accuracy and validity of ultrasonography based artificial intelligence (AI) algorithms for predicting the molecular subtypes of breast cancer patients through a meta-analysis.

**Methods:**

A search of the PubMed, Embase, Web of Science, and Cochrane Library databases was performed to locate relevant literature, and the reported studies before February 2026 were included. We evaluated the quality of the studies included by utilizing the Quality Assessment of Diagnostic Accuracy Studies (QUADAS) questionnaire. Two evaluators independently searched for literature and assessed the quality of literature included in the study.

**Results:**

A total of thirteen studies assessing a number of 13615 patients of breast cancer were included. The results demonstrated that ultrasonic imaging combined with artificial intelligence algorithm has promising accuracy and effectiveness to predict molecular subtypes of breast cancer. The pooled sensitivity and specificity were 0.89 (95%CI: 0.82-0.93) and 0.82 (95%CI: 0.77-0.86), respectively. Additionally, the diagnostic odds ratios (DOR), positive likelihood ratio (PLR) and negative likelihood ratio (NLR) were 32.10 (95%CI: 18.60-55.38), 4.84 (95%CI:3.80-6.17), and 0.14 (95%CI: 0.09-0.22). The area under the curve (AUC) was 0.91 (95%CI: 0.88-0.93). Publication bias was not significantly observed.

**Conclusions:**

Ultrasonic imaging based on artificial intelligence algorithm has good performance and application prospects for forecasting breast cancer molecular subtypes. This technique can help establish the molecular subtype of breast cancer before operation, offering effective help for the treatment plan. It may reduce unnecessary biopsy, which is anticipated to become a meaningful implement in clinical application.

**Systematic review registration:**

https://www.crd.york.ac.uk/prospero/display_record.php?ID=CRD42024599983, identifier CRD42024599983.

## 1 Introduction

Breast cancer is the second common malignant cancer in women worldwide. In 2018, a definitive diagnosis of breast cancer was made in approximately 2.1 million women (1). Moreover, there are an estimated 2.3 million new cases from 2022 onwards, accounting for 11.6% of all cancer incidence and leads to the fourth principal cause of global cancer mortality with 666,000 deaths from the disease, which accounts for 6.9% of all deaths from malignant tumors (2). In practice, breast cancer is usually classified into four different molecular subtypes including Luminal A, Luminal B, human epidermal growth factor receptor type 2 (HER2)-positive and Triple-negative breast cancer (TNBC) which in the light of expression level of estrogen receptor(ER), progesterone receptor(PR), human epidermal growth factor receptor 2(HER2), and Ki-67 (3). The luminal type has the feature of highly-expressed estrogen (ER) and progesterone (PR), and hormone therapy can be effective (4). The luminal B type, HER2- positive and TNBC are more aggressive. In addition, the HER2- positive and TNBC are more susceptible to neoadjuvant chemotherapy and have a better prognosis for achieving a pathological complete remission (pCR) (5). Therefore, early or preoperative identification of breast cancer’s molecular subtype enables timely and accurate selection of specific clinical therapies for different types of breast cancer and has a significant impact on patient outcomes and outcomes.

Currently, core needle biopsy (CNB) followed by immunohistochemical (IHC) staining serves as the clinical gold standard for molecular subtype determination. However, CNB is invasive, time-consuming, and subject to sampling error, particularly in tumors with heterogeneous composition (6). These limitations highlight the need for reliable, non-invasive approaches that can support or complement pathological diagnosis.

Although imaging examinations cannot directly determine the molecular subtype of breast cancer in the same way as pathological or molecular diagnosis, they can provide valuable assistance in suggesting the molecular subtype. As a first-line screening technique for the breast, ultrasound (US) is real-time, safe, radiation-free with low cost. It can be applied in young women during pregnancy or lactation, and can also be used as a complementary examination after mammography in women with dense breasts (7). Under different molecular subtypes, breast cancer has its own characteristics on US. Luminal cancer is mostly irregularly shaped; depleted of internal blood vessels and prominent external blood vessels and posteriorly shaded (8, 9); HER2-positive subtype is suggested by posterior microcalcifications and a mixed echogenic pattern (9). TNBC probably have benign features, for example, oval or rounded shapes, smooth margins, or ectatic margins, and have slim chance to show posterior shadowing (10, 11). The radiologist can make a characterization of the molecular subtypes of breast cancer based on the US findings on specific images of the various subtypes described above, such as the margin, morphology, calcifications, shape, and posterior echogenicity. However, there still has high inter- and intra-observer flexibility in the explanation of breast US images by radiologists. Additionally, US image artifacts during acquisition, such as noise, speckle, and signal attenuation, making it difficult for radiologists to accurately differentiate individual molecular subtypes during image recognition.

Artificial intelligence (AI) has rapidly evolved in recent years, showing significant progress and promising applications in the field of medical imaging, particularly in breast ultrasonography (US) (12). The typical workflow of AI systems includes image processing, segmentation, and feature extraction, all of which can enhance the diagnostic capabilities of imaging modalities. When integrated with breast US, AI algorithms can provide radiologists with more detailed and accurate information, potentially overcoming some of the limitations of conventional ultrasound. This integration may improve the specificity of US and offer a valuable tool for predicting the molecular subtypes of breast cancer (13, 14). While AI-based methods have shown encouraging results in this area, no meta-analysis to date has systematically assessed the overall diagnostic performance of these approaches. Therefore, we conducted a comprehensive meta-analysis to evaluate the accuracy and validity of AI algorithms applied to ultrasound imaging for the molecular subtyping of breast cancer.

## 2 Materials and methods

### 2.1 Search strategy and literature selection

Corresponding literature was searched in PubMed, Embase, Web of Science, and Cochrane Library databases, and reported studies before February 2026 were included. The study search strategy used almost all available and free Medical Subject Headings (MeSH) terms such as “breast tumor”, “breast cancer”, “breast neoplasms”, “breast carcinoma”, “ultrasound”, “ultrasonography”, “artificial intelligence”, “deep learning”, “convolutional neural network”, “machine learning”, “molecular subtypes”, “molecular subtyping”, and “triple negative breast cancer”. Two evaluators independently selected potentially matching studies based on titles and abstracts after the search was completed, and they initially screened for eligible diagnostic experimental studies by inclusion and exclusion criteria. Then the two evaluators screened the selected articles carefully by reading the full text to determine whether the studies were qualified to be included in the analysis. Regarding studies with disagreement, a third evaluator intervened to resolve issues to reach a consensus. The inclusion criteria of studies were: 1) Pathologically confirmed breast cancer patients; 2) All patients were predicted using an AI algorithm to make predictions of the corresponding molecular subtypes from breast ultrasound images; 3) The statistical indicators such as specificity and sensitivity were contained; 4) The language of the study population was limited to English. Meanwhile, the exclusion criteria of studies were: 1) Not enough data was provided to count true positive (TP), false positive (FP), false negative (FN) and true negative (TN) values; 2) Irrelevant types of studies that did not belong to cohort or case-control studies; 3) The studies contained incomplete data or did not initially explain the specific results.

### 2.2 Data extraction and quality assessment

The data of the studies which conform to the inclusion criteria have been independently collected by the two evaluators. If there is any disagreement, a third evaluator will assist and resolve the issue. Firstly, the author’s name and publication year of each study was determined. The sensitivity, specificity and the area under curve (AUC) of the AI algorithms for anticipating the molecular subtypes of breast cancer were obtained by collecting the number of patients with spreadsheets. Other information included patient age, tumor size, AI algorithm, US modality, manufacturers and selected frequency. Afterwards, two identical evaluators used the Quality Assessment of Diagnostic Accuracy Studies-2 (QUADAS-2) (15). The QUADAS-2 tool consists of four domains: patient selection, index test, reference standard, as well as flow and timing. The results of QUADAS-2 were output by the Cochrane Collaboration Network dedicated software Review Manager 5.4.

### 2.3 Statistical analysis

The analysis of all data and graphs were created using STATA 18.0 for Windows (Stata Corp, University Station, Texas, USA) with commands MIDAS and METANDI. A p value less than 0.05 indicates statistical significance. The specificity and sensitivity of the studies were collected and the TP, FP, FN and TN values were recalculated. Corresponding positive likelihood ratios (PLR) and negative likelihood ratios (NLR) were calculated based on summary receiver operating characteristic (SROC) curve which reflects the performance of diagnostic tests. To illustrate the correlation between sensitivity and specificity, symmetric or asymmetric SROC curve with 95% confidence intervals (CI) were plotted. The heterogeneity among included studies was explored using the I² statistic, which incorporates sensitivity, specificity, positive likelihood ratio, and negative likelihood ratio. If I² < 50%, we used a fixed-effects model while random-effects model was used to combine the acquired data if I² > 50%. The criteria for heterogeneity of I² were very low (0-25%), low (25%-50%), medium (50%-75%), and high (> 75%). In addition, to evaluate the bias of publication, a funnel plot was used and also conducted sensitivity analysis, which could estimate the stability of research results. Posterior probabilities were calculated using the derived PLR and NLR and Fagan plots were plotted. Fagan plot evaluates the clinical efficacy by calculating post-test probabilities for predicting molecular subtypes based on pre-test probabilities.

## 3 Results

### 3.1 Literature searches

**Figure 1:**
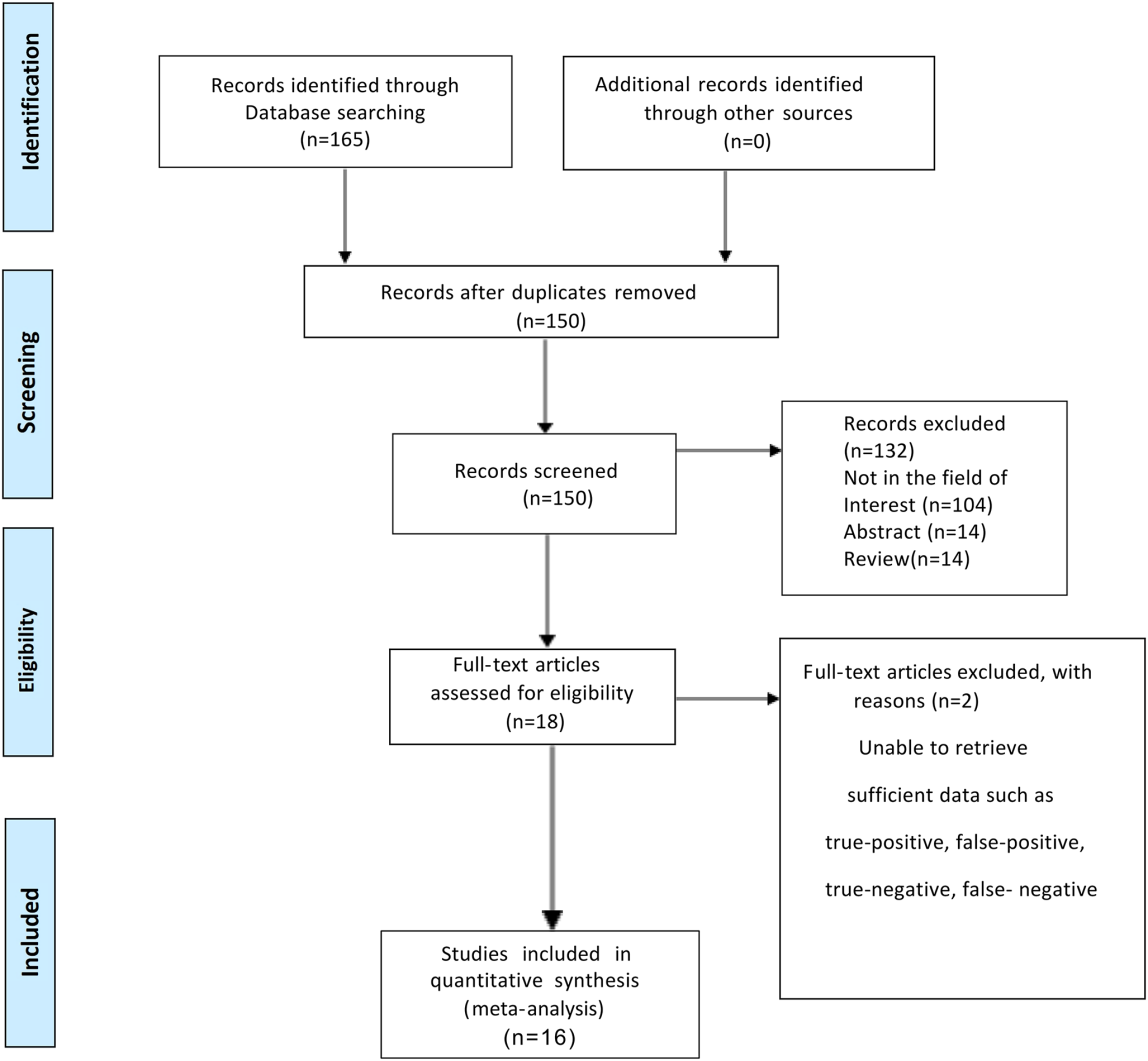
Flowchart of the study inclusion process.

After searching a systematic keyword, a number of 165 relevant literatures were first obtained. After screening the literature titles and abstracts, 15 duplicate articles and 132 articles unrelated to the research topic were excluded. One of the remaining 16 full-text articles was further excluded due to insufficient data. Finally, a total of 16 original studies including 13615 patients of breast cancer were obtained and included in this study (16–31). **Figure 1** show the detailed flowchart of the research selection procedure.

### 3.2 Characteristics of studies

The basic characteristics included in the study can be found in the **Supplementary Table 1** and the baseline characteristics of the included studies are shown in **Table 1**. Twelve studies were conducted using retrospective design, with four studies using prospective design. Three studies were multicenter studies and the remaining studies were single-center studies. Seven of the included sixteen studies used an approach employing machine learning combined with ultrasound imaging, and the remaining studies used deep learning or convolutional neural networks combined with ultrasound for the assessment of breast cancer molecular subtypes. Nine studies adopted the method of integrating multimodal data (such as combining ultrasound features with mammography features or clinical features). Nine studies provided heatmaps, and two studies provided interpretable features, including the Shapley plots, t-SNE visualizations and saliency maps. In addition, independent external validation methods were used in five studies, while the remaining studies employed internal validation techniques, such as cross-validation or random dataset splitting.

**Table 1:** Baseline characteristics of the included studies.

Author (year)	Algorithm	Validation strategy	AUC	Sensitivity	Specificity
Ma (2021)	ML	testing set	0.9	0.871	0.886
Zhang (2023) (17)	MDL-IIA	testing set	0.929	0.985	0.822
Jiang (2020) (18)	DCNN	test cohort A	0.87	0.936	0.5974
Zhang(2021) (19)	DLM	testing set	0.96	0.913	0.869
Huang (2023) (20)	DL	internal validation set	0.929	0.94	0.643
Ferrea (2023)	ML	testing set	0.824	0.818	0.742
Boulenger (2022)	CNN	testing set	0.86	0.86	0.86
Ye (2021) (23)	DCNN	testing set	0.9	0.875	0.9
Wu (2018)	ML	testing set	0.88	0.8696	0.8291
Zhou (2021) (25)	ACNN	test cohort A	0.962	1	0.91
Xu (2023) (26)	ML	training set	0.917	0.771	0.898
Wu (2022) (27)	ML	training set	0.832	0.8531	0.8049
Gong (2023) (28)	ML	training set	0.96	0.465	0.816
Wang (2025) (29)	DLRN	testing set	0.924	0.96	0.767
Liu (2024) (30)	DCNN	testing set	0.969	0.827	0.8738
Zhang (2025) (31)	ML	testing set	0.823	0.717	0.789

AUC, area under the receiver operating characteristic curve; ACNN, Assembled convolutional neural network; CNN, Convolutional neural network; DLM, Deep learning model; DCNN, Deep convolutional neural network; ML, machine learning; MDL-IIA, Multi-modal deep learning with intra- and inter-modality attention modules; DLRN, Deep learning radiomics integrated model.

### 3.3 Data quality assessment

**Figure 2:**
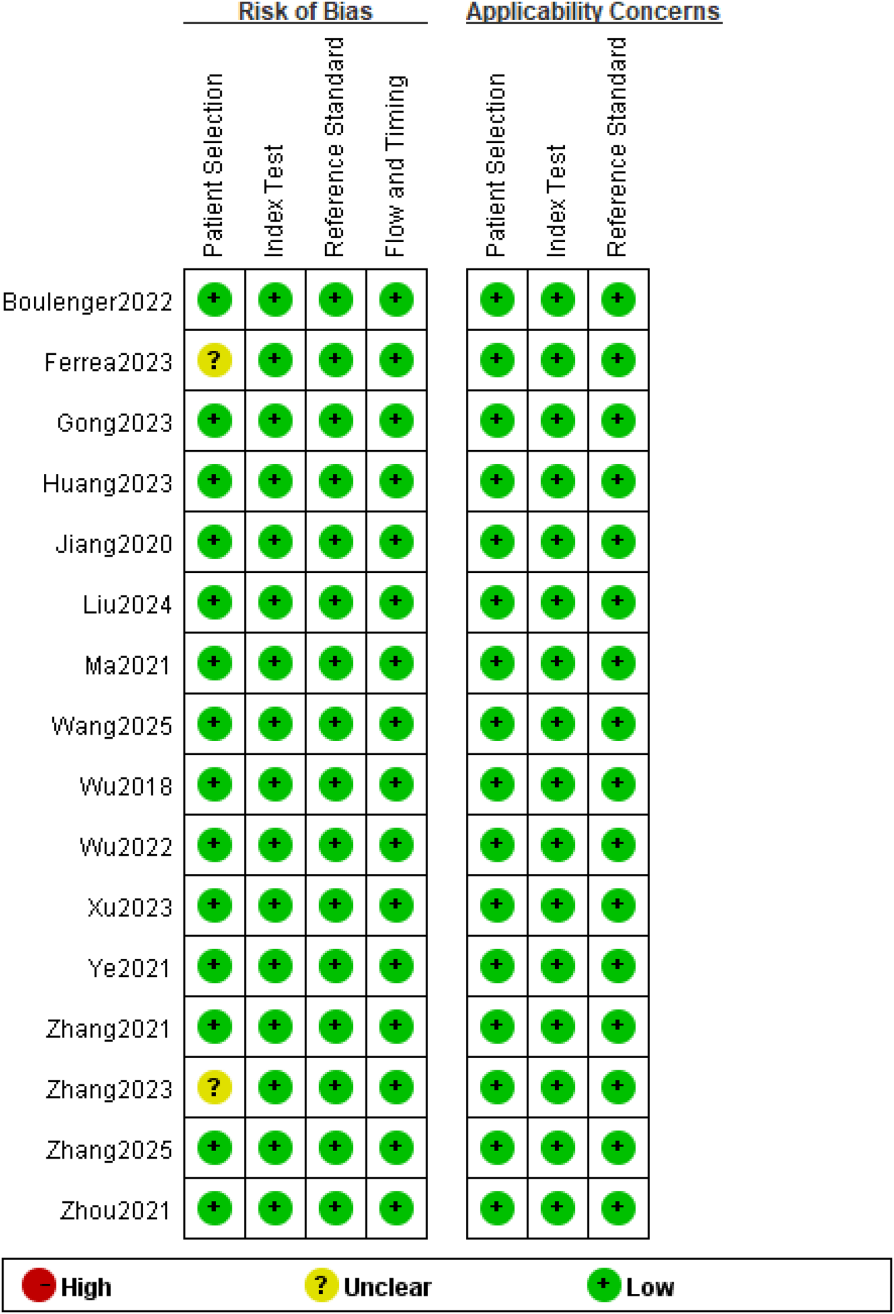
Methodologic quality of the included studies assessed according to the Quality Assessment of Diagnostic Accuracy Study 2 tool for risk of bias and applicability concerns.

**Figure 2** indicates the results of the QUADAS-2. Two studies showed an unclear risk of bias in the “Patient Selection” domain due to the lack of specific description on how patients are enrolled. The failure to adequately clarify whether consecutive or randomized patient samples were used raised concerns about potential selection bias. All studies showed a minimal risk of bias within the domains of “Index Test”. All selected studies used biopsy and/or histopathology as reference standards, therefore the risk of bias in the “Reference Standard” field was low. Additionally, in the “Flow and Timing” domain, all patients were included in the study and did not show a significant high risk of bias. Therefore, the methodological evaluation based on the QUADAS-2 checklist concluded that the included studies’ quality was high. It also identified that most of the quality evaluation items did not have a significantly higher risk of bias.

### 3.4 Data analysis

**Figure 3:**
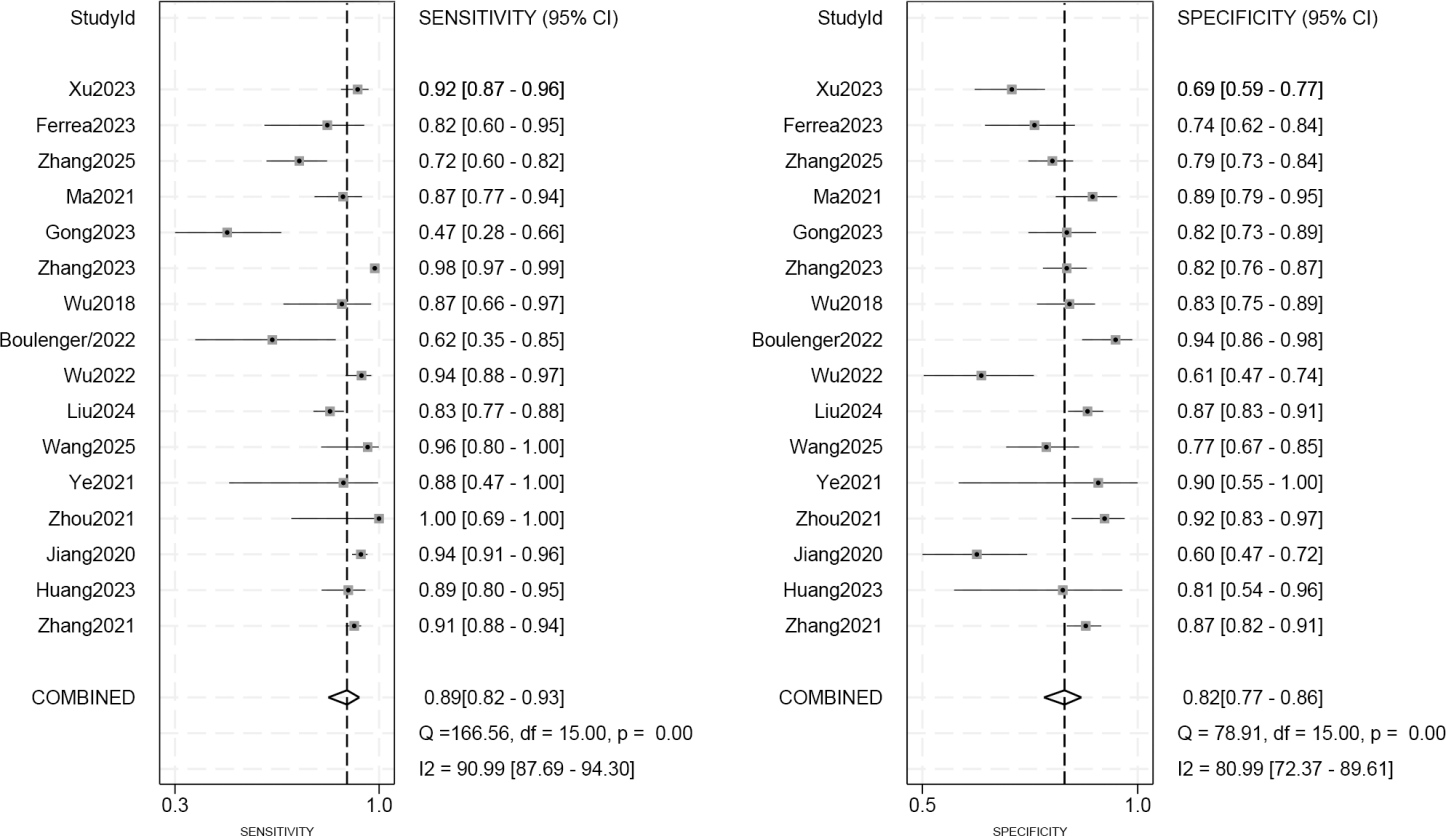
Forest plot of ultrasound imaging-based prediction of molecular subtypes in breast cancer patients using AI algorithms.

**Figure 4:**
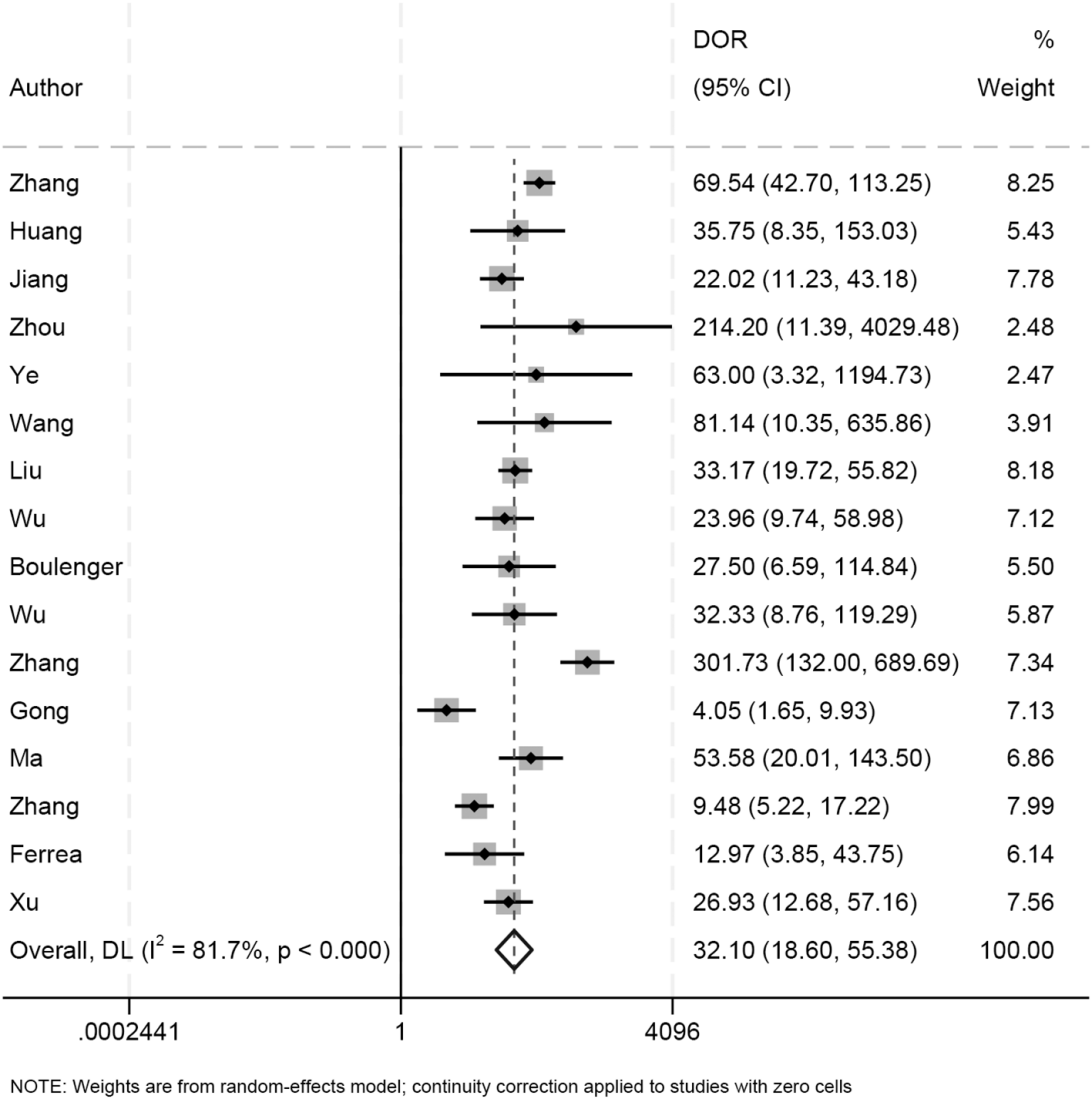
The Diagnostic Odds Ratios (DOR) for predicting molecular subtypes of breast cancer.

**Figure 5:**
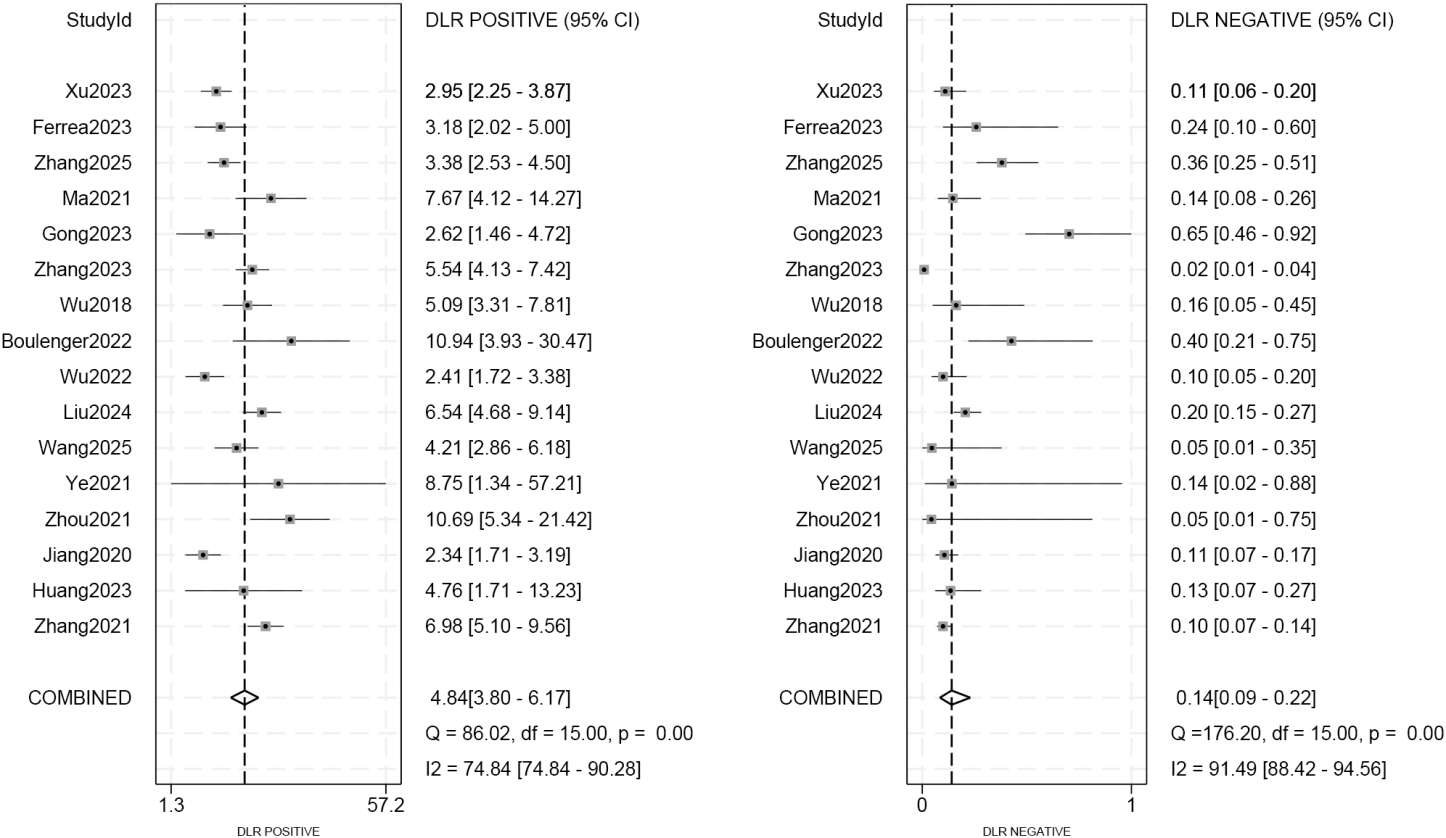
The positive and negative diagnostic likelihood ratios (DLR) for predicting molecular subtypes of breast cancer.

**Figure 6:**
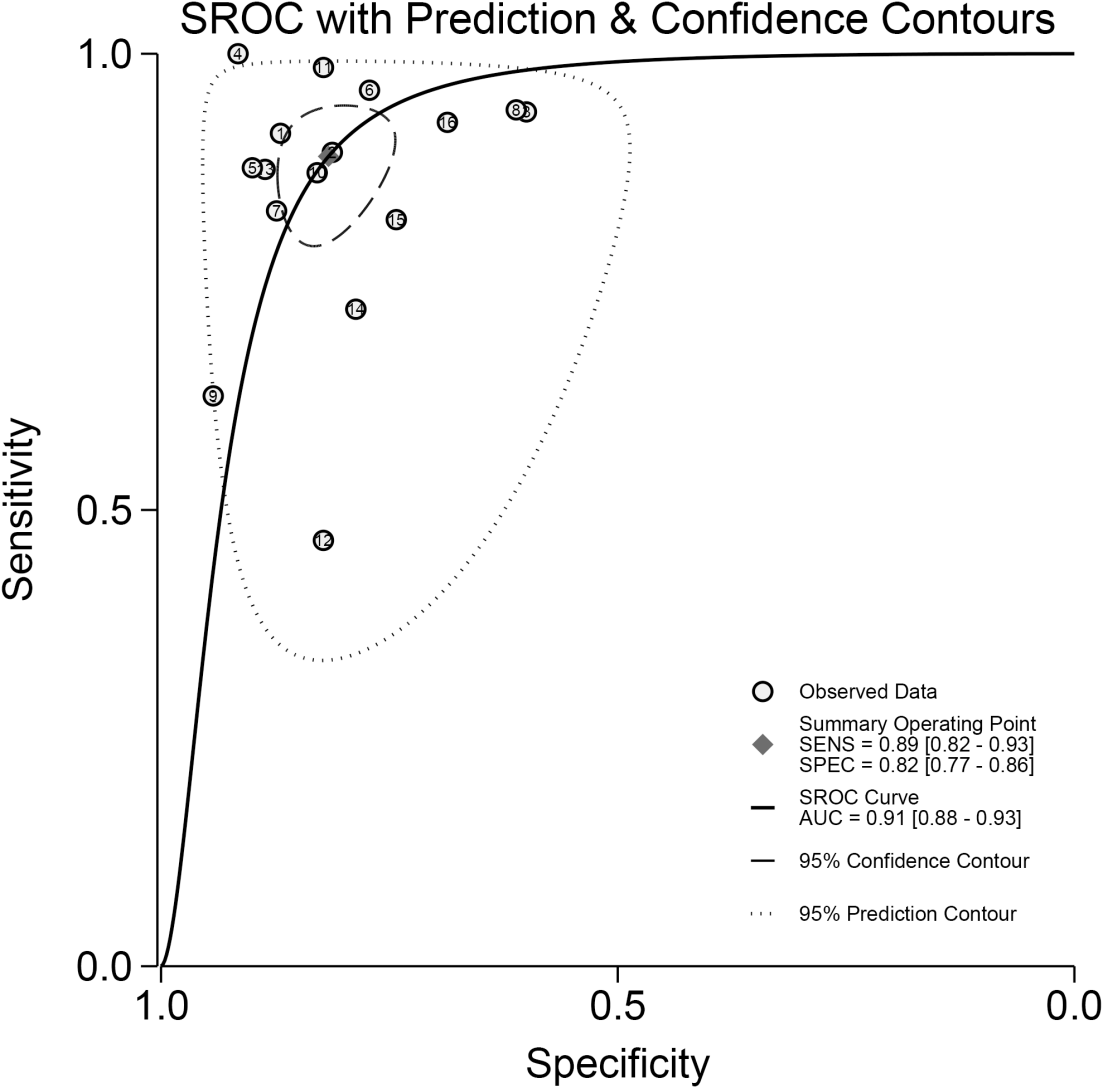
The Summary receiver operating characteristics (SROC) curve. Illustrating sensitivity versus specificity for diagnostic studies,with observed data,a summary operating point, SROC curve,95 percent confidence and prediction contours,and an area under the curve of 0.91.

**Figure 7:**
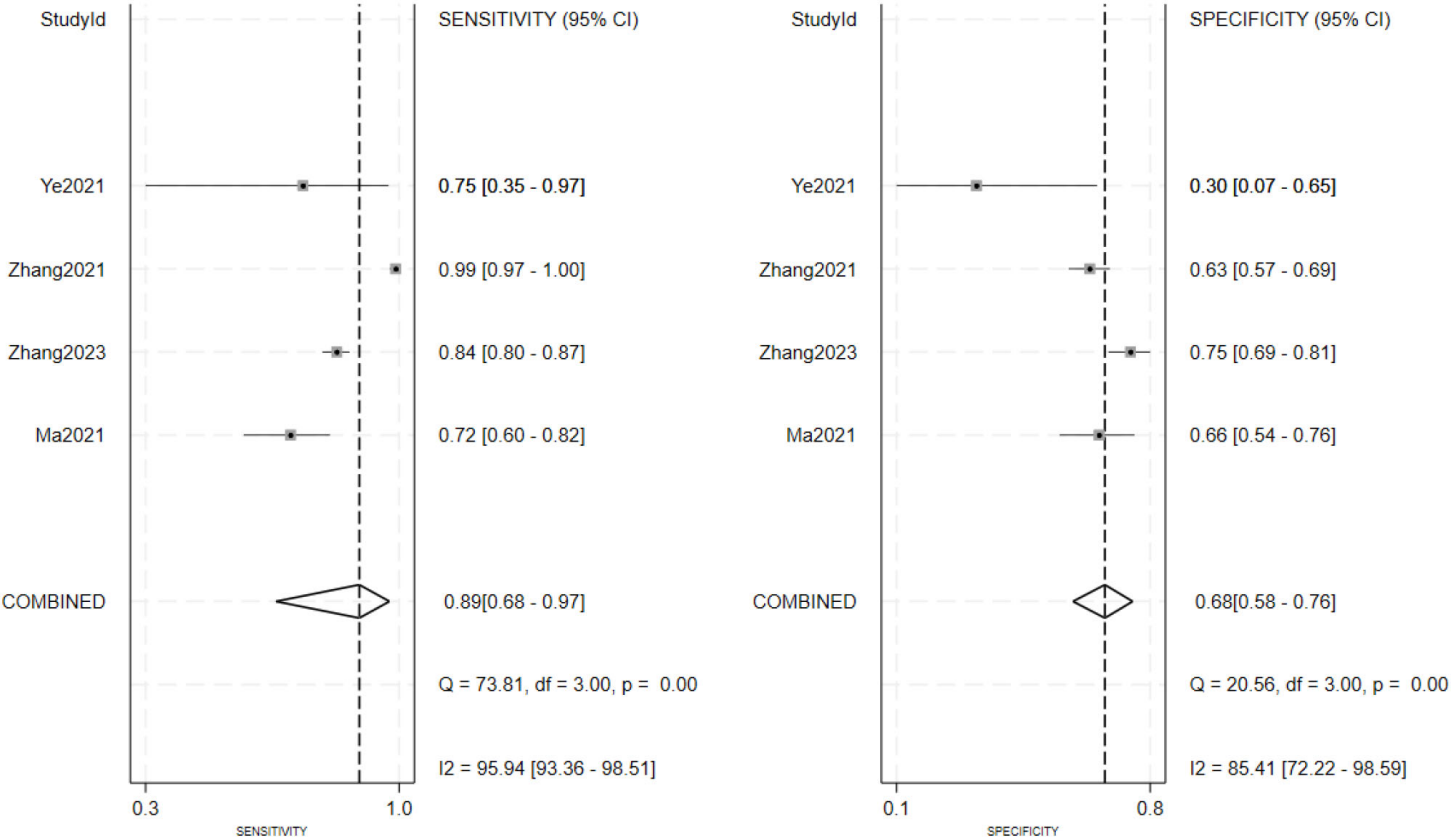
Forest plot of radiologists in four studies diagnosing molecular subtypes of breast cancer. Combined sensitivity is 0.89 and combined specificity is 0.68.

As seen in **Figure 3**, the sensitivity and specificity of the ultrasound model using AI for forecasting the molecular subtypes of breast cancer were 0.89 (95%CI: 0.82-0.93; p< 0.01; I²=90.99%) and 0.82 (95%CI: 0.77-0.86; p<0.01; I²=80.99%). Due to its high heterogeneity, sensitivity and specificity were selected to be combined in a random effects model. And the diagnostic odds ratios (DOR), positive likelihood ratio (PLR) and negative likelihood ratio (NLR) merged by the random effects model were 32.10 (95%CI: 18.60-55.38), 4.84 (95%CI:3.80-6.17), and 0.14 (95%CI: 0.09-0.22) (**Figures 4**, **5**), respectively. The area under the cure (AUC) was 0.91 (95%CI: 0.88-0.93) (**Figure 6**). After merging the random effects model, I²=81.7% was obtained, indicating that this study has a high heterogeneity. In addition, we collected and counted four studies that used radiologists rather than AI technology to diagnose the molecular subtypes of breast cancer (**Figure 7**). Among them, the obtained sensitivity was 0.89 (95% CI:0.68-0.97), specificity was 0.68 (95% CI:0.58-0.76) and AUC was 0.74 (95% CI:0.70-0.78). The sensitivity analysis helped to exclude studies with potential bias one by one and the results did not show significant changes.

Due to the observed high heterogeneity in the studies, we conducted subgroup analysis based on the research, focusing on Data source, research type, Model, Algorithm, verification method, and sample size (**Table 2**). According to subgroup analysis results, only algorithm classification demonstrated statistical significance. Among these, Deep Learning (DL) exhibited higher sensitivity (0.95 vs 0.84 vs 0.84) than Machine Learning (ML) and Convolutional Neural Network (CNN) (p < 0.05). In addition, the pooled DOR from internal validation studies was 4.17 (95% CI: 3.28–5.29), while the pooled DOR from external validation studies was 5.51 (95% CI: 2.72–11.16). Although the point estimate was slightly higher in the external validation subgroup, the difference between the two groups was not statistically significant (p>0.05). Notably, the external validation subgroup exhibited extremely high internal heterogeneity (I²=87.0%, p < 0.001) with a wide confidence interval range, reflecting substantial variability in existing external validation findings and uncertainty inherent in small-sample subgroup analyses. To further investigate the sources of inter-study heterogeneity (I²=81.7%, p<0.01), we employed meta-regression analysis. First, a null model (Model 0) was established to assess baseline heterogeneity levels. Subsequently, three models were constructed by progressively incorporating potential influencing factors: Model 1 included algorithm type (with ML algorithms as the reference, and DL and CNN as dummy variables); Model 2 added log-transformed sample size to Model 1; Model 3 further incorporated study design characteristics (multicenter, external validation). Model selection was based on the adjusted R² (Adj R², representing the proportion of heterogeneity explained), overall model significance (p−value of the F−test), and the principle of parsimony. All analyses were estimated using the restricted maximum likelihood (REML) method, with the Knapp–Hartung modification applied to control for small−sample bias. **Table 3** presents the results of the meta-regression model comparison. The null model revealed high heterogeneity (I²=81.7%, τ²=0.8324). Model 1, which included only algorithm type, explained 51.44% of the heterogeneity (Adj R²=51.44%), and the model as a whole was significant (F = 5.43, p=0.019). Specifically, DL algorithms demonstrated significantly higher diagnostic accuracy compared to ML algorithms (β=1.678, 95% CI: 0.577–2.778, p=0.006), while CNN algorithms showed no significant difference from ML. Model 2 showed a slight decrease in explanatory power after adding log-transformed sample size (Adj R² = 49.84%), and the sample size effect was not significant (p=0.466). Model 3, incorporating all variables, further decreased explanatory power to 41.04%, and the model as a whole was not significant (p=0.141). Based on explanatory power and parsimony, Model 1 was determined to be the optimal model, indicating that algorithm type is the primary factor influencing diagnostic accuracy.

**Table 2:** The performance evaluation of the subgroups.

Subgroup	No.ofstudies	Sensitivity	Specificity	PLR	NLR	DOR
Datasource						
Single-center	13	0.88 (0.80-0.93)	0.83 (0.78-0.87)	5.06 (3.95-6.48)	0.14 (0.08-0.25)	4.63 (3.63-5.90)
Multicenter	3	0.92 (0.89-0.94)	0.67 (0.57-0.76)	2.85 (2.10-3.87)	0.11 (0.07-0.16)	3.26 (1.68-6.33)
Studydesign						
Retrospective	11	0.91 (0.88-0.93)	0.82 (0.77-0.87)	5.04 (3.81-6.67)	0.14 (0.08-0.22)	4.66 (3.56-6.11)
Prospective	5	0.90 (0.70-0.97)	0.80 (0.69-0.88)	4.49 (2.75-7.34)	0.12 (0.04-0.43)	3.97 (2.41-6.55)
Modelcomposition						
binary	7	0.89 (0.83-0.94)	0.82 (0.71-0.89)	4.86 (3.05-7.75)	0.13 (0.08-0.21)	4.27 (2.86-6.39)
multi	9	0.90 (0.78-0.96)	0.82 (0.77-0.87)	5.08 (3.82-6.76)	0.12 (0.06-0.27)	4.60 (3.40-6.22)
Classificationofalgorithms						
ML	7	0.84 (0.72-0.91)	0.77 (0.71-0.83)	3.72 (2.99-4.62)	0.21 (0.12-0.37)	3.44 (2.70-4.38)
DL	4	0.95 (0.89-0.98)	0.83 (0.79-0.87)	5.92 (4.75-7.37)	0.05 (0.02-0.12)	5.54 (4.41-6.95)
CNN	5	0.84 (0.74-0.91)	0.87 (0.74-0.94)	6.40 (3.40-12.05)	0.18 (0.12-0.29)	6.28 (3.05-12.91)
Validationtype						
Internal	11	0.87 (0.77-0.93)	0.81 (0.76-0.86)	4.60 (3.61-5.87)	0.16 (0.09-0.29)	4.17 (3.28-5.29)
External	5	0.92 (0.89-0.94)	0.84 (0.71-0.91)	5.67 (3.11-10.33)	0.10 (0.08-0.13)	5.51 (2.72-11.16)
Samplesize						
<100	5	0.84 (0.74-0.91)	0.88 (0.78-0.93)	6.8 (3.72-12.44)	0.18 (0.10-0.31)	6.34 (3.33-12.08)
100-400	7	0.86 (0.73-0.93)	0.78 (0.72-0.83)	3.87 (3.09-4.83)	0.18 (0.10-0.35)	3.59 (2.81-4.58)
>400	4	0.94 (0.86-0.97)	0.81 (0.71-0.89)	5.04 (3.19-7.96)	0.08 (0.03-0.18)	4.93 (3.00-8.08)

PLR, positive likelihood ratio; NLR, negative likelihood ratio; DOR, diagnostic odds ratio; ML, machine learning; DL, deep learning; CNN, Convolutional neural network; All results were calculated using a random effects model.

**Table 3:** Meta-regression results: predictors of effect size variability.

Model	Variables included	Tau²	*I²*(%)	Adj R² (%)	F-value	p-value
model0	Intercept only	0.832	81.7	–	–	–
model1	Algorithm type (DL, CNN vs. ML)	0.404	63.3	51.44	5.43	0.01
model2	Algorithm type + log(sample size)	0.417	65.3	49.84	3.72	0.04
model3	Algorithm + log(sample size) + multicenter + external validation	0.49	65.8	41.04	2.15	0.14

ML, machine learning; DL, deep learning; CNN, Convolutional neural network; Tau² represents the estimated between-study variance; Adj R² indicates the proportion of between-study variance explained by the model covariates; Model 0 serves as the null model without predictors; Model 1 included algorithm type alone (with ML algorithms as the reference, and DL and CNN as dummy variables); Model 2 added log-transformed sample size to Model 1; Model 3 further incorporated study design characteristics (multicenter, external validation).

### 3.5 Fagan plot analysis and publication bias

**Figure 8:**
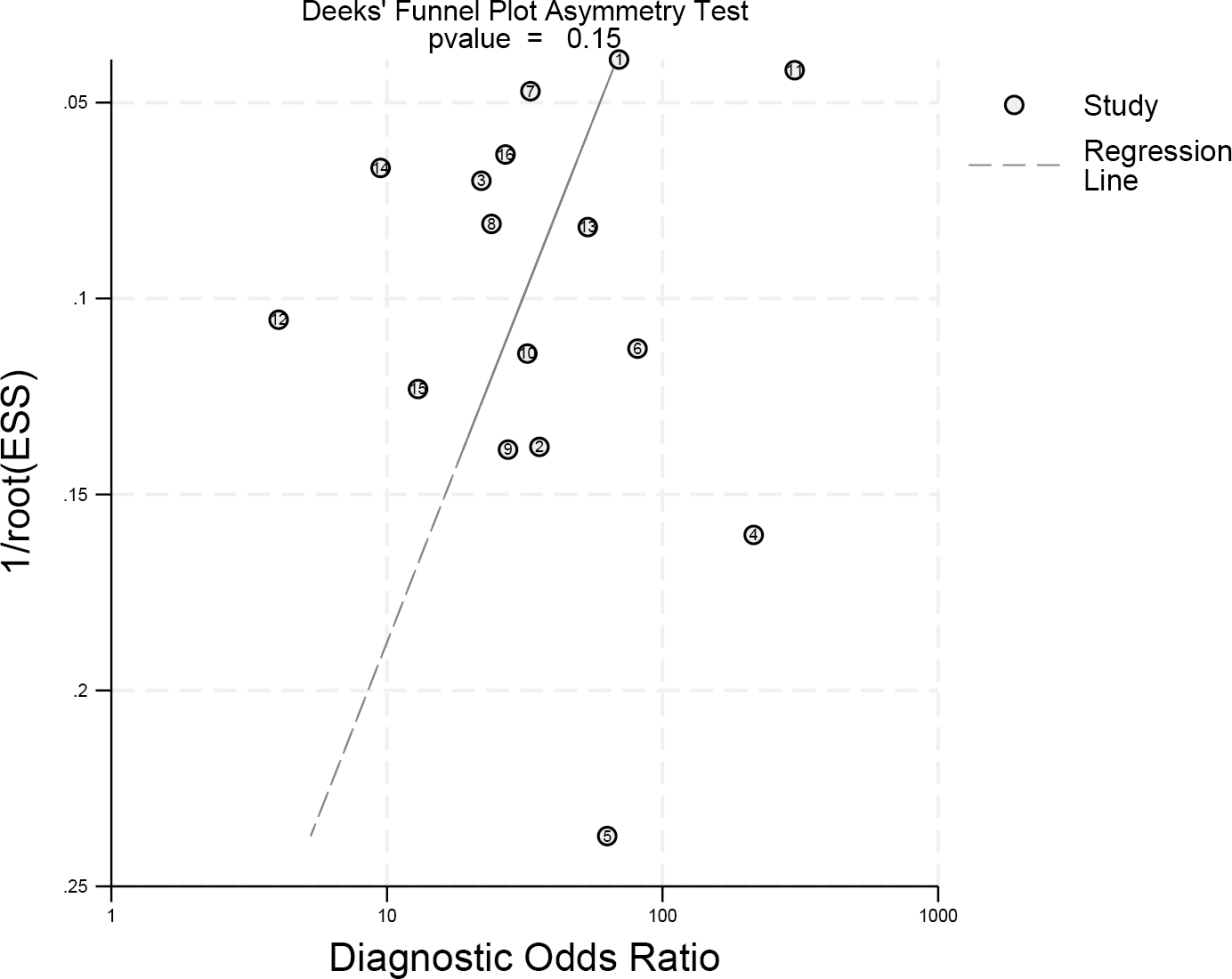
The Deeks funnel plot asymmetry test to determine publication bias in the literature evaluation (p = 0.15) indicated there was no obvious publication bias. Each dot represents an individual study.

Fagan diagram analysis showed that the AI algorithm could provide some valuable information for the determination of molecular subtypes of breast cancer. Calculated PLR and NLR values by setting prior probabilities were 25%, 50%, and 75%, respectively. As shown in the **Figure 8**, the probability of the AI algorithm correctly predicting the molecular subtype of breast cancer was 83% under the condition of the pre-test probability was 50% and after the “positive” measurement, and the probability of the AI algorithm dropped to 12% after the “negative” measurement. When the prior probabilities were 25% and 75%, the positive pre-test probabilities were 62% and 94%, the negative pre-test probabilities were 4% and 29%, respectively (**Figures 1**, **2**).

**Figure 9:**
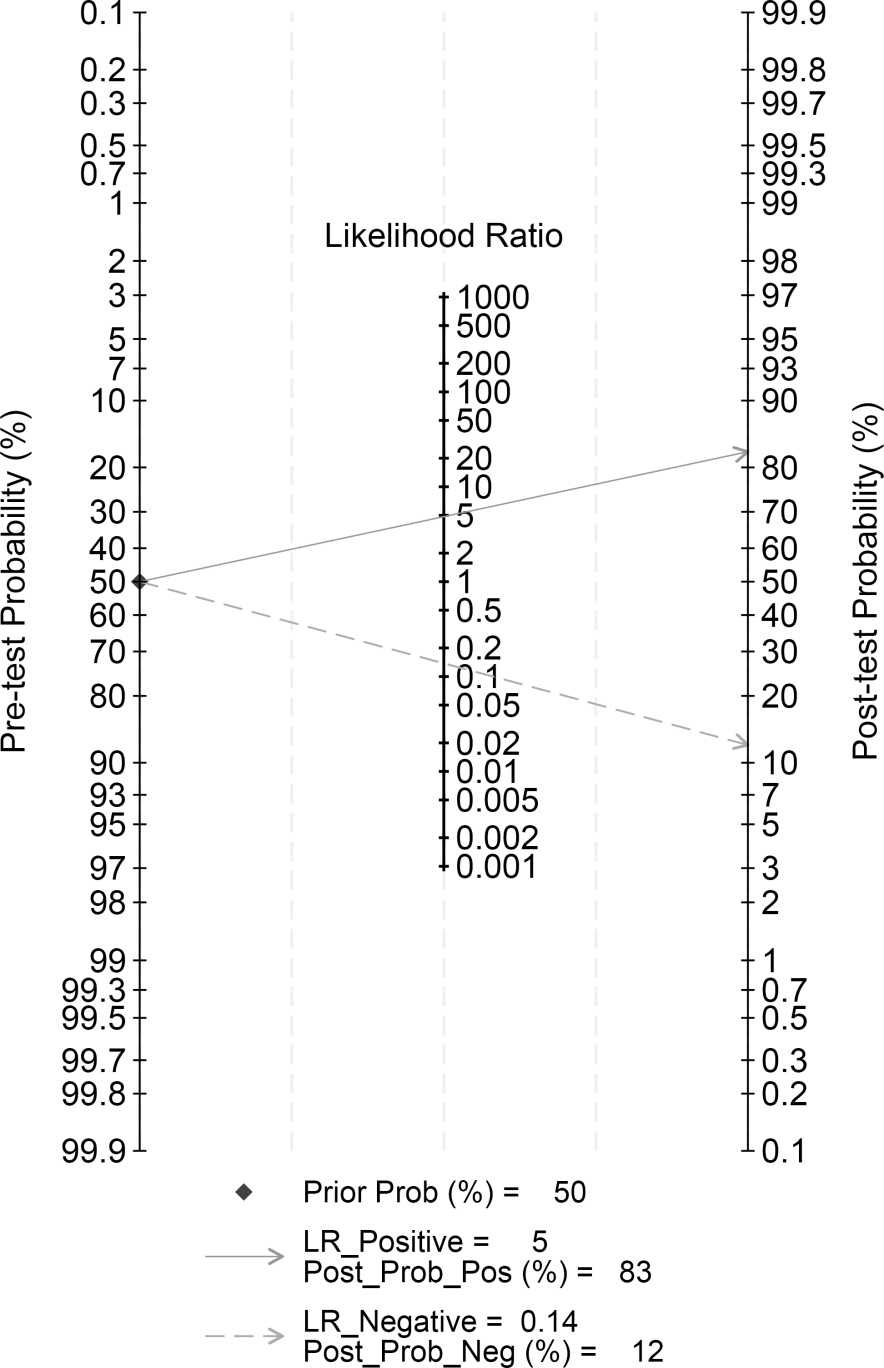
Fagan plot depicting the probability of predicting molecular subtypes in breast cancer patients, based on ultrasound imaging using AI algorithms. The Fagan plot is composed of the left vertical axis representing the pre-test probability, the middle vertical axis representing the likelihood ratio, and the right vertical axis representing the post-test probability.

The publication bias of the included study can be obtained through the DEEK’ plot (**Figure 9**). The funnel plot asymmetry test showed no clear publication bias (P = 0.15).

## 4 Discussion

Defining the molecular subtype-specific molecular features of breast cancer has significance for selecting clinically individualized treatment plans, which could improve the prognosis and living standards of patients. As an alternative to the subjective interpretation of ultrasound imaging, which relies on the empirical judgment of radiologists, AI algorithms introduce automation and standardization. Moreover, AI algorithms enable more meticulous observation of subtle features, thereby reducing subjective errors associated with human interpretation and missed diagnosis. Therefore, this study focuses on the application of AI algorithms based on single or multiple ultrasound imaging model for prediction of molecular subtypes of breast cancer for meta-analysis. Sixteen studies were selected for comprehensive analysis and used to assess the feasibility of AI algorithms in this field. By analyzing the selected studies, the pooled AUC of the included studies is 0.91 (95%CI: 0.88-0.93) that we concluded ultrasound imaging utilizing AI algorithms demonstrates high accuracy and credibility in forecasting breast cancer molecular subtypes. This represents that AI algorithms may help radiologists to confirm the molecular subtypes of breast cancer, as well as to serve as a reminder or reference to avoid unnecessary misdiagnosis and omission.

Molecular subtypes can have a significant impact on the initiation and metastasis of cancer. HER2-positive patients are more common than Luminal A patients in multifocal tumors and lymphovascular invasion (32). Luminal breast cancer usually has bone metastases, while HER2-positive and TNBC are more prone to brain and lung metastases, and TNBC has a poorer prognosis after brain metastasis (33). In the treatment of different molecular subtypes, luminal breast cancer demonstrates favorable responses to endocrine therapy, such as tamoxifen. For HER2-positive breast cancer, anti-HER2 targeted therapy—including agents like trastuzumab and pertuzumab—forms the cornerstone of treatment. In contrast, chemotherapy (e.g., anthracyclines, taxanes, and cyclophosphamide) remains the primary approach for TNBC. Moreover, HER2-positive and TNBC are also more effective in preoperative chemotherapy than other types of breast cancer, while Luminal breast cancer is more suitable for postoperative radiotherapy (34, 35). Although pathological diagnosis serves as the gold standard for differentiating molecular subtypes of breast cancer, immunohistochemical staining using CNB has corresponding limitations. To overcome these limitations, several studies have combined breast imaging techniques with AI to identify molecular subtypes of breast cancer. Among our selected studies that include multimodal model, Zhou et al. (25) used an assembled convolutional neural network (ACNN) model has achieved better diagnostic efficiency than CNB before surgery (AUC: 0.89 vs. 0.67, p<0.05), and the ACNN model has made 7 additional correct predictions compared with CNB (31 vs24 out of 37 cases). The ACNN model achieved an AUC of 0.89, sensitivity of 92.31%, and specificity of 80.00% in predicting the four-class molecular subtypes of breast cancer. Moreover, the model demonstrated even better performance in distinguishing TNBC from non-TNBC, yielding an AUC of 0.970 (95% CI: 0.936, 1.000). These results indicate that the model exhibits robust diagnostic efficacy for both four-class classification and TNBC prediction. Zhang et al. (17) established a multi-modal deep learning model with intra- and inter-modality attention modules (MDL-IIA) by combining mammography and ultrasound imaging to forecast breast cancer molecular subtypes. This model achieved an accuracy of 88.5% (95% CI: 86.0%, 90.9%), a Matthews correlation coefficient (MCC) of 0.837 (95% CI: 0.803, 0.870), and an AUC of 0.929 (95% CI: 0.903, 0.951) in distinguishing luminal and non-luminal subtypes. Beyond these established subtypes, the recent recognition of “Low HER2” (HER2-low) expression has refined the traditional binary HER2 classification and carries significant therapeutic implications (36). HER2-low is defined as tumors with HER2 IHC scores of 1+ or 2+ without gene amplification on *in situ* hybridization (ISH), historically categorized as HER2-negative. This group constitutes a substantial proportion (approximately 45-55%) of all breast cancers, spanning both hormone receptor-positive and TNBC subtypes (37). Critically, the advent of novel antibody-drug conjugates (ADCs), such as trastuzumab deruxtecan (T-DXd), has demonstrated remarkable efficacy in treating HER2-low metastatic breast cancer, effectively creating a new, therapeutically actionable category (38). This paradigm shift underscores the limitations of conventional classification systems in the era of precision oncology and highlights the urgent need for AI studies to move beyond traditional binary or four-class frameworks. Future research should aim to develop models capable of identifying this “Low HER2” phenotype non-invasively, as it represents a vast patient population newly eligible for targeted therapy.

As a non-invasive screening method, AI-based breast imaging has significant technical differences compared to tissue biopsy. Screening techniques using AI can potentially reduce the burden on patients physically, psychologically, and financially. Accordingly, it can also reduce misdiagnosis or missed diagnoses caused by physician fatigue or overwork. Meanwhile, the performance exhibited by different AI algorithms in the research we selected also varies. In the study using ML algorithm, Gong et al. (28) achieved the highest sensitivity and AUC, which were 0.875 (80.4%, 92.3%) and 0.953 (0.935, 0.967), respectively in the study using CNN algorithm, although Boulenger et al. (22) showed lower sensitivity than Gong et al. (28) (0.86 vs 0.875), they performed better in specificity (0.86 vs 0.802). This phenomenon aligns with our research findings. Our subgroup analysis and meta-regression analysis indicate that algorithm type is a key source of heterogeneity, with deep learning typically outperforming machine learning. This can be attributed to deep learning’ ability to automatically learn hierarchical features from raw data, thereby circumventing the labor-intensive and subjective manual feature engineering required by machine learning. Although DL models exhibit stronger learning capacity, they are more sensitive to data distribution, and generalizing beyond the training domain remains a significant challenge (39, 40). Additionally, the “black-box” nature of deep learning models, particularly CNNs, presents profound and multi-faceted challenges. Unlike traditional ML, where feature importance can often be traced, DL’ opaque, high-dimensional representations create an epistemological gap between data-driven prediction and mechanistic understanding, challenging the principles of evidence-based reasoning. This profound lack of transparency significantly hinders clinical uptake, as medical practitioners are unable to incorporate opaque outputs within their decision-making processes, undermining confidence and clinical independence (41). Furthermore, it creates a significant ethical and accountability vacuum, complicating responsibility in cases of error. Explainable AI (XAI) methods aim to overcome the opacity of black-box models and render transparent the decision-making processes of AI systems (42). Among the 16 studies included in our review, 11 implemented XAI approaches; of these, nine provided heatmaps, while two offered other interpretable features such as Shapley plots, t-SNE visualizations and saliency maps. Nonetheless, five studies did not provide any form of AI interpretability method. In systematic reviews of AI-based studies, it is increasingly recommended to assess whether included research provides such interpretable outputs. Future studies should incorporate XAI techniques to enhance model transparency and facilitate clinical translation.

The sensitivity and specificity obtained from this study were 0.89 (95%CI: 0.82-0.93) and 0.82 (95%CI: 0.77-0.86), respectively. In several studies investigating AI for cancer diagnosis, researchers have compared the performance of AI models against radiologists, providing preliminary insights regarding its potential role. Li et al. (43) constructed an ultrasound-based deep convolutional neural network (DCNN) model for diagnosing thyroid cancer. The DCNN model achieved high accuracy of 89.8%, which surpassed the radiologists with the accuracy of 78.8%. Similarly, studies by Wang et al. (44) and He et al. (45) found their respective CNN models attained accuracy superior to that of comparing radiologists in specific diagnostic tasks. It is crucial to interpret these findings with caution; direct comparisons are often conducted in controlled research settings and may not fully replicate the complexity of generalized clinical practice. Moreover, the number of such head-to-head comparison studies in our review is limited, and their statistical power to support a universal claim of AI “surpassing” human experts is insufficient. A more nuanced and clinically relevant perspective is to examine how AI can augment radiologists’ performance. Consistent with the notion of augmentation, evidence from our reviewed studies highlights AI’s potential to complement and enhance clinical decision-making. Zhang et al. (19) demonstrated that while a deep learning model (DLM) had slightly lower sensitivity than the BI-RADS assessment by radiologists, its significantly higher specificity and accuracy suggested it could aid in reducing unnecessary biopsies by mitigating false-positive calls. Their analysis provided empirical support for this potential: the DLM correctly reclassified 70.76% of patients assessed as BI-RADS 4a (low suspicion for malignancy) by radiologists as benign, achieving a diagnostic accuracy of 92.86% in this subgroup and suggesting a potential reduction in unnecessary biopsies by 67.86%, with a false negative rate of 10.4%. However, while such findings from individual studies are promising, a comprehensive assessment of clinical utility and the net benefit of implementing AI-assisted biopsy triage requires formal decision-analytic frameworks, such as Decision Curve Analysis (DCA). More directly, Ma et al. (16) evaluated a decision tree (DT) model as an assistive tool. Their results showed that when radiologists were assisted by the DT model, their diagnostic sensitivity, specificity, and accuracy all improved. Notably, the performance gain was more pronounced for junior radiologists (increases of 0.090, 0.125, and 0.114, respectively) than for senior radiologists (increases of 0.060, 0.090, and 0.083). Therefore, based on the current evidence, a more measured conclusion is that AI models show promise as effective tools to augment radiologists’ diagnostic capabilities, rather than to universally “surpass” them. The integrated analysis of radiologist expertise and AI can potentially improve diagnostic outcomes by leveraging the strengths of both: AI’s consistency in processing quantitative imaging patterns and the radiologist’s contextual, clinical judgment (46). Crucially, this synergistic effect is particularly pronounced in improving the performance of trainees and junior practitioners, helping bridge the experience gap and reduce diagnostic variability. Future research should prioritize rigorous prospective studies that directly evaluate AI’s impact on clinical workflows and patient outcomes in real-world settings, moving beyond simple accuracy comparisons. Reports should include clinical utility metrics such as decision curve analysis, threshold probability, and net benefit.

Beyond ultrasound, mammography and magnetic resonance imaging (MRI) are pivotal modalities in breast cancer management, each with distinct strengths and limitations. Mammography is the cornerstone of population-based screening but has reduced sensitivity in dense breasts. MRI offers superior soft-tissue contrast and spatial resolution but is costly and less accessible. In contrast, ultrasound provides a real-time, radiation-free, and cost-effective alternative, particularly valuable for evaluating dense breast tissue and guiding interventions. However, ultrasound is operator-dependent, exhibits inter-observer variability, and has limited sensitivity for microcalcifications. Adding ultrasound testing after mammography can greatly increase the detection rate of breast cancer in women with dense breast and increased risk of cancer (47). In recent years, the integration of multi-modal data represents a frontier for enhancing diagnostic precision. Each imaging modality captures unique and complementary aspects of tumor biology—mammography detects microcalcifications, ultrasound reveals morphological and hemodynamic features and clinical parameters further enrich the diagnostic context. Combining these complementary data sources allows AI models to construct a more holistic tumor profile. Among the studies included in our meta-analysis, nine adopted such a multimodal approach, integrating different modalities such as ultrasound and mammography, clinical features, or contrast-enhanced ultrasound. Gong et al. (28) constructed a predictive model combining conventional ultrasound and contrast-enhanced ultrasound radiomics. Compared to the conventional ultrasound and single contrast-enhanced ultrasound models, the combined radiomics model achieved an AUC of 0.953 (95% CI: 0.935, 0.967) in this study. It has been demonstrated that contrast-enhanced ultrasound with tumor blood perfusion can further improve the diagnostic performance of ultrasound radiomics models. These findings reveal the key: future high-performance AI systems will no longer rely on a single data stream, but will be based on the intelligent fusion of multimodal information. This approach echoes the clinical decision-making process of radiologists, which involves integrating multiple sources of information. The great challenges faced by multimodal AI include constructing large-scale, accurately registered datasets, developing robust fusion architectures (such as attention mechanisms), and demonstrating its clinical added value through rigorous validation (48). Future research should prioritize the development of standardized multimodal data acquisition and interpretable fusion strategies to fully unlock the potential of integrated diagnosis in personalized breast cancer diagnosis and treatment.

In recent years, the field of breast cancer artificial intelligence has witnessed a series of state-of-the-art (SOTA) achievements in architecture and training strategies (49–51). Chaotic Learning Rate Scheduling (CLRS) achieved 93.91% accuracy on the BUSI ultrasound dataset using the EfficientNetV2-Small model by replacing traditional cosine annealing with chaotic dynamics via logical mapping, demonstrating that optimizing training dynamics yields significant improvements. Further demonstrated through LayerCAM visualization, CLRS enhances lesion-focused attention through its optimized training strategy. InceptionNeXt-Transformer innovatively fuses convolutional neural networks with visual transformers through a four-stage decoupled design, achieving high-precision generalization across seven datasets encompassing histopathology, mammography, and ultrasound (100% accuracy for histopathology binary classification, 99.97% for mammography, 92.86% accuracy for ultrasound), demonstrating the synergistic potential of multi-scale modeling and cross-modal attention; A systematic comparative study encompassing nearly 30 CNN and ViT models further reveals significant modality dependence—CNN-based architectures like EfficientNetV2-Small excel in ultrasound (90.52% accuracy), while multi-scale ViT (MViTv2-Base) and lightweight CNNs perform best in pathology (91.67% accuracy). Outperformed in ultrasound diagnosis (90.52% accuracy), while multi-scale ViT (MViTv2-Base) and lightweight CNNs achieved the best results in histopathology diagnosis (91.67% accuracy). In contrast to these high-performance SOTA reports, the effects observed in this meta-analysis generally fell below the peak values reported in SOTA studies. This performance gap can be attributed to multiple factors: (a) Most included studies employed classical CNN architectures with simple feature concatenation, lacking advanced fusion strategies such as cross-modal attention or decoupled multi-scale modeling; (b) Training hyperparameters (e.g., learning rate scheduling) were rarely optimized, preventing models from reaching their full potential. Future research urgently requires direct comparisons of cutting-edge architectures in large multicenter cohorts, alongside standardized reporting guidelines covering architecture design, training strategies, and interpretability assessments. These initiatives are crucial for bridging the gap between laboratory-level performance and real-world efficacy.

It is worth noting that this diagnostic meta-analysis also has limitations. Firstly, we only included published articles, unpublished research or grey literature was not incorporated. Although we have chosen a large search scope and implemented multiple screening measures in our literature search, potential publication bias may still exist. This may lead to an overestimation of the effect size and potentially yield false-positive conclusions, thereby compromising the accuracy of the findings. Even though mainstream databases such as PubMed, Embase, Web of Science, and the Cochrane Library cover the majority of high-quality research worldwide, this selection may still introduce language bias. Consequently, our analysis may not encompass all relevant evidence, potentially affecting the comprehensiveness and generalizability of results (e.g., pooled diagnostic performance metrics). Future systematic reviews should consider collaborating with researchers proficient in relevant languages or employing broader search strategies to minimize such bias as much as possible. Secondly, the predominance of retrospective study designs among the included literature, while common in initial exploratory phases of AI research, may limit the direct assessment of real-world clinical performance and generalizability. The strength of our conclusions would be enhanced by future prospective, multi-center trials that minimize selection bias and more closely mimic routine clinical workflow. Finally, only a minority of included studies (5/16) reported external validation results, limiting the generalizability of this study’ conclusions to unseen data. Although our subgroup analysis did not reveal statistically significant differences in diagnostic accuracy between internal and external validation studies (p=0.463), high heterogeneity and wide confidence intervals within the external validation subgroup indicate that the existing evidence remains unstable. Models validated internally carry risks associated with overfitting and optimistic bias, and significant variability may exist across different external validation cohorts. Therefore, we recommend prioritizing high-quality external validation studies to rigorously assess the model’ generalizability in real-world clinical settings.

## 5 Conclusion

In summary, our findings provide robust evidence supporting the value and potential of integrating ultrasound imaging with AI algorithms for predicting breast cancer molecular subtypes. This non-invasive approach may serve as a valuable supplement to core needle biopsy, reducing patient discomfort and procedural risks. As an auxiliary tool characterized by considerable potential, it could assist clinicians in developing more personalized and effective treatment strategies for patients, thereby improving their prognosis. However, the findings should be interpreted with caution due to the limited number of studies, significant heterogeneity, predominance of retrospective designs, and variability in imaging and AI methodologies. Further large-scale, prospective, multicenter studies with standardized imaging protocols and rigorous external validation are required to confirm the robustness and generalizability of current evidence.

## Data Availability

The original contributions presented in the study are included in the article/**Supplementary Material**. Further inquiries can be directed to the corresponding authors.
