# Supplementary material for: The value of artificial intelligence in ultrasound imaging for predicting molecular subtypes of breast cancer: a meta-analysis
Source: Front Oncol. 2026 Mar 12;16:1748473. doi: 10.3389/fonc.2026.1748473 (PMC13017395; doi:10.3389/fonc.2026.1748473)

Supplementary Material

# Supplementary Figures and Tables

| Author(year) | Design | Data source | Country | Patients | Age(years±SD) | Size（mm） | AUC | Sensitivity | Specificity |
| --- | --- | --- | --- | --- | --- | --- | --- | --- | --- |
| Ma(2021) | Retrospective | Single-center | China | 600 | 47.59±11.37 | - | 0.9 | 0.871 | 0.886 |
| Zhang(2023) | Retrospective | Single-center | China | 3360 | (54±8)training(54±10)test | 19.9±10.4 | 0.929 | 0.985 | 0.822 |
| Jiang(2020) | Retrospective | Multicenter | China | 1275 | - | 19(15–22) | 0.87 | 0.936 | 0.5974 |
| Zhang(2021) | Retrospective | Single-center | China | 2542 | - | - | 0.96 | 0.913 | 0.869 |
| Huang(2023) | Prospective | Multicenter | China | 603 | - | 23(6–50) | 0.929 | 0.94 | 0.643 |
| Ferrea(2023) | Retrospective | Single-center | Canada | 88 | 52±14 | 27.0±11.6 | 0.824 | 0.818 | 0.742 |
| Boulenger(2022) | Retrospective | Single-center | China | 831 | 49.40±10.15(validation)50.93±10.07(test) | - | 0.86 | 0.86 | 0.86 |
| Ye(2021) | Retrospective | Single-center | China | 1446 | - | - | 0.9 | 0.875 | 0.9 |
| Wu(2018) | Retrospective | Single-center | China | 140 | 51.45±9.39 | - | 0.88 | 0.8696 | 0.8291 |
| Zhou(2021) | Prospective | Multicenter | China | 807 | 59.30±12.75 | - | 0.962 | 1 | 0.91 |
| Xu(2023) | Retrospective | Single-center | China | 342 | - | - | 0.917 | 0.771 | 0.898 |
| Wu(2022) | Retrospective | Single-center | China | 264 | 53.13 ± 11.25 | 22.48 ± 10.01 | 0.832 | 0.8531 | 0.8049 |
| Gong(2023) | Prospective | Single-center | China | 166 | 51.56 ± 10.33 | - | 0.953 | 0.875 | 0.802 |
|  |  |  |  |  |  |  |  |  |  |

## Supplementary Figures

**Supplementary Figure 1.**


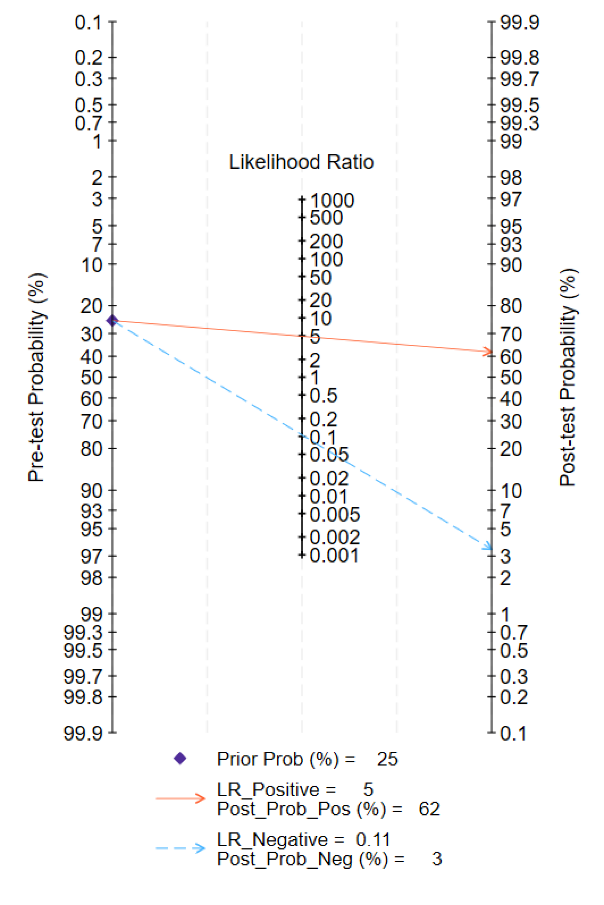


**Supplementary Figure 2.**


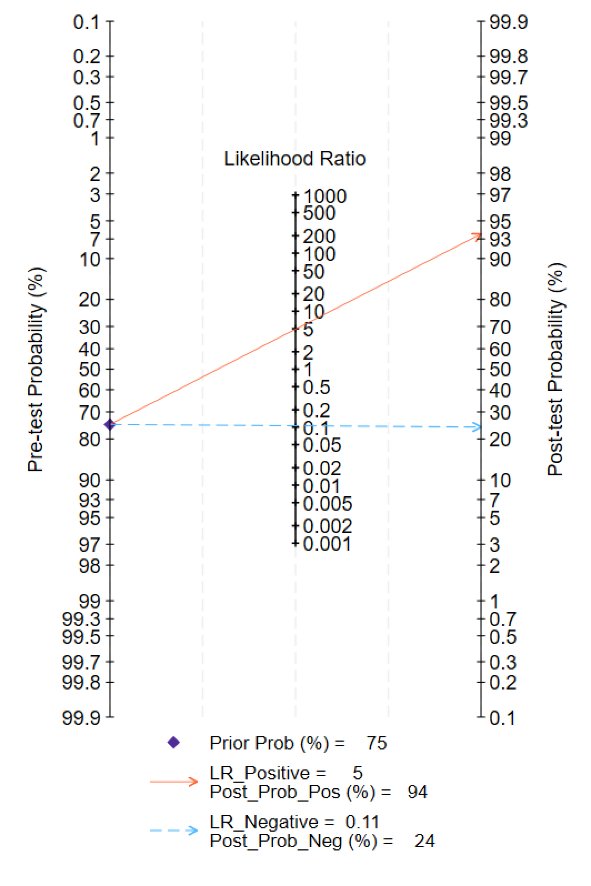

Supplement: Supplementary file 1 [file DataSheet1.docx]
